# The Artificial Intelligence in Teledermatology: A Narrative Review on Opportunities, Perspectives, and Bottlenecks

**DOI:** 10.3390/ijerph20105810

**Published:** 2023-05-12

**Authors:** Daniele Giansanti

**Affiliations:** Centre Tisp, Istituto Superiore di Sanità, 00161 Rome, Italy; daniele.giansanti@iss.it; Tel.: +39-06-49902701

**Keywords:** telemedicine, mobile health, remote monitoring digital solutions, dermatology, medical device, teledermatology, App, self-care, artificial intelligence, survey

## Abstract

Artificial intelligence (AI) is recently seeing significant advances in teledermatology (TD), also thanks to the developments that have taken place during the COVID-19 pandemic. In the last two years, there was an important development of studies that focused on opportunities, perspectives, and problems in this field. The topic is very important because the telemedicine and AI applied to dermatology have the opportunity to improve both the quality of healthcare for citizens and the workflow of healthcare professionals. This study conducted an overview on the opportunities, the perspectives, and the problems related to the integration of TD with AI. The methodology of this review, following a standardized checklist, was based on: (I) a search of PubMed and Scopus and (II) an eligibility assessment, using parameters with five levels of score. The outcome highlighted that applications of this integration have been identified in various skin pathologies and in quality control, both in *eHealth* and *mHealth*. Many of these applications are based on Apps used by citizens in *mHealth* for self-care with new opportunities but also open questions. A generalized enthusiasm has been registered regarding the opportunities and general perspectives on improving the quality of care, optimizing the healthcare processes, minimizing costs, reducing the stress in the healthcare facilities, and in making citizens, now at the center, more satisfied. However, critical issues have emerged related to: (a) the need to improve the process of diffusion of the Apps in the hands of citizens, with better design, validation, standardization, and cybersecurity; (b) the need for better attention paid to medico-legal and ethical issues; and (c) the need for the stabilization of international and national regulations. Targeted agreement initiatives, such as position statements, guidelines, and/or consensus initiatives, are needed to ensure a better result for all, along with the design of both specific plans and shared workflows.

## 1. Introduction

### 1.1. Background

The application of the principles of telemedicine to dermatology is referred to as teledermatology. Historically, teledermatology (TD) has been classified into *real-time* (RT) TD and *store-and-forward* (SaF) TD [1]. SaF TD allows a patient to contact the dermatologists through asynchronous consultations; this reduces the wait time to have a meeting. RT TD is also frequently used in dermatology, using video channels for the interaction between the patient and the doctors.

Since 1995, there has been a pseudo-exponential growth in the use of TD. It is documented by the very high scientific production trend in this area. The number of publications is well over one thousand to date [2]. Its use has an important impact on the perceived quality of the service delivery. It has the potential to improve the relationship between the patient and the *health domain* in terms of monitoring, treatment, and the process itself. This can lead to an increase in the satisfaction of both the patient [3] and of the other actors involved (e.g., general practitioners, specialists, assistants [4]). The COVID-19 pandemic demonstrated that TD could be an accessible, accurate, and cost-effective substitute for conventional *face-to-face* dermatological consultations [5]. Artificial intelligence (AI) has also proven to be, together with other technologies, a far-reaching technological solution during the pandemic [6]. In particular, AI has shown to be of great support in the dermatological field during the pandemic [7].

The combination of AI with TD can bring a synergistic benefit to the practice of dermatology.

TD, on the one hand, thanks to digital health solutions (e.g., *eHealth* and *mHealth*), can reduce distances, avoid travel, and bring treatment directly to the patient’s home.

AI, on the other hand, is using increasingly performing algorithms, developed by data science experts. Both AI and TD have advantages from the availability of Big Data. They are useful both for training and for large volumes of digital data access. AI is presenting itself as a technological opportunity with the potential to improve the clinical process, the decision making, and the workflow in dermatology practice.

These considerations lead us to believe that among the challenges and perspectives of dermatology we precisely find the embedding of AI into TD [8]. This is why it is important to focus efforts and initiatives in this area.

Other studies on the introduction of AI in the digital sharing processes of diagnostic imaging in other sectors of the *health domain* have shown a very recent scientific interest. They focused both on the development opportunities and prospects and on the bottlenecks [9,10]. In fact, in addition to the development of algorithms, when facing integration into healthcare, it is necessary to address a series of issues (e.g., acceptance, regulations, standardization, safety, workflows) involving different professional figures. This is happening also in other fields of imaging.

In digital radiology, for example, challenges and opportunities of integration with AI have been highlighted in several studies [11,12], together with important emerging problems [13,14].

Further, in digital pathology, similar studies are also facing the opportunities and problems [14,15,16,17,18].

In view of the rapid technological innovations in the *health domain*, it is strategic to address the same issue in TD as well.

### 1.2. Problems, Research Question, and Purpose of the Study

The scientific dissemination of TD and AI is available starting from 2018 [19]. Two papers have been published in the years 2018 and 2019, while the other ones are from the year 2020 up to the present. The interest in this topic is therefore quite recent [19]. The topic is very important because applying telemedicine and AI to dermatology could improve both the quality of healthcare of citizens and the workflow of healthcare professionals. This introduction in the *health domain* must address many issues, ranging from the development of algorithms to the regulations implemented. A *narrative review* has been proposed in this study as a practical tool to face this relatively young sector.

The aim of the *narrative review* is to provide a comprehensive overview of the current state of the field, identify opportunities and challenges for integration, and provide guidance for clinicians, researchers, and policymakers interested in advancing this area of healthcare.

The specific aims are, in detail:

- To assess the trends and the evolution of the studies in this field;
- To assess the current state of the art methods in teledermatology and artificial intelligence, including their strengths and limitations;
- To identify the potential benefits of integrating teledermatology with artificial intelligence, such as increased accuracy and efficiency in diagnosing skin conditions, to improve patient outcomes and prioritize cost savings;
- To explore the challenges of and barriers to integrating teledermatology with artificial intelligence, such as data privacy concerns, regulatory issues, and the need for specialized expertise;
- To provide guidance on best practices for implementing and using teledermatology and artificial intelligence in dermatology, including recommendations for data collection and management, model development and validation, and clinical decision-making.

Overall, the subjects addressed in the studies provide a useful idea for both future research directions and, indirectly, to address the current gaps and bottleneck.

## 2. Methods

The study was based on a *narrative review*. In this *narrative review*, after having defined the research question and sub-questions in the aims, we decided to follow: (a) a standardized checklist for narrative reviews and (b) a properly defined process of review.

The process of review, reported in the algorithm below, comprehended the development of a search strategy.

– The definition of a process of selection;
– The assessment of the study quality;
– A data analysis/synthesis identifying the emerging patterns.

This *narrative review* used a standardized checklist designed for the category of narrative reviews (ANDJ Narrative Checklist).

It is available online at [20]. The algorithm was the following:

*Algorithm*

1. Set the search query to: “(teledermatology [Title/Abstract]) AND (artificial intelligence [Title/Abstract])”;
2. Conduct a targeted search on PubMed and Scopus using the search query from step 1;
3. Exclude conference papers from the search results;
4. Select studies published in peer-reviewed journals that focus on experiences of integration of artificial intelligence with teledermatology;

5. For each study, evaluate the following parameters:
  - N1: Is the rationale for the study in the introduction clear?
  - N2: Is the design of the work appropriate?
  - N3: Are the methods described clearly?
  - N4: Are the results presented clearly?
  - N5: Are the conclusions based and justified by results?
  - N6: Did the authors disclose all the conflicts of interest?

6. Assign a graded score to parameters N1–N5, ranging from 1 (minimum) to 5 (maximum);
7. For parameter N6, assign a binary assessment of “Yes” or “No” to indicate if the authors disclosed all the conflicts of interest;

8. Preselect studies that meet the following criteria:
  - Parameter N6 must be “Yes”;
  - Parameters N1–N5 must have a score greater than 3;

9. Include the preselected studies in the overview.

## 3. Results

### 3.1. Preliminary Considerations on the Results

All the studies returned by the algorithm had the parameter N6 with “*Yes*” and the parameters N1–N5 with a score > 3.

The search [19] provided 36 components [8,21,22,23,24,25,26,27,28,29,30,31,32,33,34,35,36,37,38,39,40,41,42,43,44,45,46,47,48,49,50,51,52,53,54] at the date of this study.

In detail, the components were arranged into four groups:

(1) Twenty-two scientific papers [8,21,22,23,24,25,26,27,28,29,30,31,32,33,34,35,36,37,38,39,40];
(2) One randomized controlled trial [41];
(3) Twelve reviews [42,43,44,45,47,48,49,50,51,52,53,54];
(4) One systematic review [46].

The scientific dissemination is available starting from year 2018. Two papers have been published in the years 2018 and 2019; 34 papers (94.45%) have been published from the year 2020 up to the present. It is notable that the increase in the number of publications started with the outbreak of the COVID-19 pandemic. All this highlights that this is a very young scientific sector.

All this shows that the topic is very recent and is receiving significant attention, and that TD and AI, integrated, are offering themselves as an important resource in dermatology, in a wide range diagnosis applications, from skin cancer lesions to sun damage perilesions [23,41].

We decided to analyze *papers* and *reviews* separately.

Separating the analysis of scientific papers from the reviews is a logical and sensible approach. The reason behind this is that scientific papers and reviews serve different purposes and have distinct characteristics. Scientific papers are typically written by researchers who conduct specific experiments or studies to generate new data or medical knowledge (MK). The analysis of scientific papers, therefore, tends to be more focused on the specific research question, methodology, and data analysis, with a strict angle and focus on the research problem being investigated. On the other hand, reviews are broader and consolidate fields by analyzing and synthesizing a large body of the literature to identify patterns, trends, and gaps in the MK. Reviews may cover multiple studies, experiments, or research areas and draw conclusions based on the analysis of the collective evidence. Authors of reviews may also critique the research methods and findings of individual studies, but their main focus is on providing an overview of the field as a whole. Therefore, by separating the analysis of scientific papers from reviews, it is easier to maintain a clear and concise focus on the specific research question being investigated in the scientific papers, while also providing a comprehensive overview of the field as a whole through the reviews. This approach can help ensure that both the specific details and the broader context of the research are properly addressed and understood, especially in this relatively young sector.

### 3.2. Data Syntesys of the Overview of Scientific Papers

The analysis suggests a possible categorization according to the following emerging fields of interest detected in the scientific papers:

- *The opportunities and the perspectives.* Those studies that have mostly focused on the horizons of the application of TD and AI are included here;
- *The role of the tool and the devices.* The studies that have addressed the topic by focusing, in particular, on the tool dedicated to AI are included here;
- *The applications in quality control*. The studies that have addressed the applications of AI in quality control in diagnostic imaging are included here;
- *The integration in mHealth*. The studies that have addressed the integration in *mHealth* (where recently self-diagnosis applied to telemonitoring is gaining ground) are included here;
- *The integration in the health domain: the acceptance, the standardization, and the management issues*. All the studies that have somehow dealt with the aspects related to the integration of the health domain are included here (i.e., those studies that have offered a look at the problems of integration in stable healthcare processes). This topic is broad and includes, for example, studies on accuracy, acceptance, and regulatory and organizational aspects.
- Some studies, in addition to having primarily dealt with one of the issues identified above, have also dealt with other issues that overlap in a secondary way.

Table 1 reports a sketch of the data synthesis of the overview of scientific papers.

#### 3.2.1. The Opportunities and the Perspectives

This theme has been touched upon, albeit sometimes in a nuanced way, by all 23 papers [8,21,22,23,24,25,26,27,28,29,30,31,32,33,34,35,36,37,38,39,40,41]. Generally, all the studies converge on the opportunities and general perspectives of the TD and AI in improving the quality of one or more services/applications in the *health domain*.

Three studies have focused on identifying in a specific way the opportunities and perspectives that TD and AI seem to have in the middle and long term [8,21,22].

Liopoys et al. [8], highlighted the important prospects of AI in this area, noting that thanks to the characteristics of learning from imaging obtained in dermoscopy, great opportunities are expected in the field of diagnosis of skin lesions, which thanks to TD can even be performed remotely. In this direction, we also find the studies by Li et al. [21] and by Mahmood [22]. According to Li et al. [21], recently, there are three key elements to consider in TD and AI: there has been a clarification of the role and expectations of AI in TD; there has been an expansion of the applicability of imaging techniques; and the developments of TD have been completed in many applications. According to their predictions, these three elements will have the ability both to offer great help in the *health domain* and to define routine models. Mahmood et al. [22] recognized important developments in this sector with a leading role in prognosticating dermatoses and, of course, in the three stages of analysis articulated in *classification, detection and diagnosis*. The authors foresaw important prospects in *mHealth* in virtual diagnosis and in the research and development of increasingly advanced machine learning models.

#### 3.2.2. The Role of the Tools and Devices

According to six studies, an important role in this area is played by the tools and devices [23,24,25,26,30,31]. Four studies investigated this in a specific way [23,24,25,26].

Maqsood and Damaševičius [23] proposed a unified CAD model approach using a proposed AI structure capable of providing both the lesion segmenting and classifying at the level of the skin. They showed that their tool achieved high performance with regard to several imaging parameters, including comprehending the accuracy. Escalé-Besa et al. [24] proposed a project addressing the use of AI as a support tool, with the aim of providing an outcome on the AI effectiveness and limitations, highlighting that external testing is essential for regulating the tools in the development of machine learning models in the settings of primary care. Lee et al. [25] revisited the important steps in the evolution of the tools that can be used in TD and AI, highlighting how, in this area, together with new technologies, new approaches and methodologies must coexist with historically consolidated methods. Jain A et al. [26] remarked that many cases in dermatology are often initially valued by non-dermatologists, as with, for example, nurse practitioners or primary care specialists. They proposed an automatic tool based on TD and AI that demonstrated an improvement of the diagnosis by these professionals with a high potential for improving the quality of healthcare in dermatology.

Two studies, which focused on the category dedicated to *mHealth* [30,31], indirectly addressed the importance of wearable devices based on smartphones and Apps installed therein as a secondary aspect.

#### 3.2.3. The Applications in Quality Control

Aspects related to the application of AI in the quality control of the images have been specifically addressed in four studies [27,28,29]. Quality is a crucial parameter for an effective and efficient TD consultation. The application of AI seems to be, in these studies, promising.

Jaloboi et al. [27] introduced a tool based on AI implementing a quality assessment based on a convolutional neural network approach capable of identifying criticalities in the image quality. The tool demonstrated quality detection performances similar to that of dermatologists allowing for the improvement of the TD process. Montilla et al. [28], in line with the previous study, focused on the topic of quality assessment in TD and demonstrated that AI has a leading role in ensuring the usefulness of a certain image in TD. Mayer et al. [29], also facing the field of quality assessment in TD, proposed a comparative analysis among different AI solutions for the quality assessment, highlighting the importance to tune an AI model for a specific TD application. In addition, Henandez Montilla et al. [28] faced the field of the Dermatology Image Quality Assessment and discussed how AI could be useful to ensure the clinical utility of images for remote consultations and clinical trials.

#### 3.2.4. The Applications in mHealth

Two studies particularly focused on the application of TD and AI in *mHealth*. The study by Hadeler et al. [30] was entirely focused on this topic with reference to clinical research. The study highlighted that, thanks to the boost of research during the pandemic, there has been a great boom in this sector. According to the authors, the use of mobile applications demonstrated the potential to improve the experience of study subjects and physicians and to increase the pool of individuals willing to participate in research beyond the pandemic. However, there were *pros* and *cons* that must be taken into consideration together with system components that are essential. Po et al. [31] also focused entirely on *mHealth* and addressed user satisfaction in the *health domain*. They proposed a study on the assessment of user satisfaction with a smartphone-compatible application using an AI algorithm designed for the assessment of pigmented lesions. The results highlighted both that the parameters related to user satisfaction were high and that these Apps based on AI solutions were promising and have potential for all the actors within the *health domain*. However, it should be noted that all the other 21 studies [8,21,22,23,24,25,26,27,28,29,32,33,34,35,36,37,38,39,40,41] unanimously consider the application in mHealth of TD and AI as a promising future.

#### 3.2.5. Towards the Integration in the Health Domain: The Acceptance, the Standardization, and the Management Issues

Nine studies have specifically focused on particular aspects of the *integration in the health domain*, a core aspect to comprehending the issues of *standardization, acceptance, performance analysis, management aspects* and any other related ones [32,33,34,35,36,37,38,39,40].

Sun et al. [32,33] proposed a study divided into two parts which addressed the critical issues of the use of TD and AI with reference also to standardization. Taken together, the two studies delivered two important and strategic scientific messages:

(a) Ref. [32] Image post-processing capabilities vary greatly based on the user and the intended function. Technology standards are not always implemented or reported. Critical issues are also detected on the consent procedure and on other aspects of privacy and data confidentiality;
(b) Ref. [33] The choices of different technical applications of image acquisition are hardly ever proposed in the tools. Users of these applications should consider which tools have standardized functionalities capable of improving the image quality.

The problem of the use of standards was also addressed in a study conducted by Lewinson et al. [34] with specific reference to the reality of the Canadian nation.

Some studies have evaluated *acceptance* through surveys [35,36]. The survey reported in [35] highlighted the belief that, after COVID-19, these methods could be of aid, though it did not replace but instead supported professionals; thus, specific regulatory measures were needed. Jones et al. [36] based on a survey concluded that the technological innovations, including the use of AI and TD, could contribute to improving and enlarging the use of dermoscopy among general practitioners.

The accuracy and performance in relation to TD and AI under different perspectives have been investigated in [37,38,39]. In the study by Muñoz-López et al. [37], an AI algorithm was evaluated in a telemedicine setting configured for TD where patients provided images via telemedicine. The study demonstrated that this configuration of the healthcare process was promising both in the triage and in the assessment of skin lesions. MacLellan et al. [38] demonstrated that a specific tool had very high specificity and sensitivity in TD and AI applications, showing potential to assist the clinical decision-making process. From another perspective, Papachristou and Bosanquet, by carrying out a study comparing the accuracy of two countries in the national screenings within a certain dermatological field [39], deduced that TD and AI could aid in speeding up the early diagnosis of melanoma, making the process easier and more economical without stressing the structures of the health systems.

The issue of process improvements in management terms has also been addressed in a project proposed by Felmingham et al. [40].

Felmingham et al. [40] have proposed a study, now in progress, with the aim to: (a) compare the decision taken by an AI-based classifier with that of the teledermatologist, the histopathologist, and other key professional figures. (b) Consequently, the study aims to assess the impact on diagnostic and process management decisions including time, costs, personnel, and activities.

Two studies, which focused on the category dedicated to mHealth [30,31], indirectly addressed the aspects of integration in the health domain, in terms of problems to overcome [30] and acceptance [31].

### 3.3. Data Syntesys of the Overview of the Reviews

It is important to analyze the point of view of the reviews in this area, as in all the relatively young research areas, in order to also have a measure of progress of the specific research, together with the development of related topics.

In this specific case, it can be observed that the selected reviews did not have the specific task of addressing the development of the integration of TD and AI jointly, which we find were handled as a non-primary sub-research topic. Taking this into account, it is reported that: four reviews addressed the issue by relating it to the impact of the COVID-19 epidemic [42,43,44,46], also with specific reference to some national realities [46]. Some studies have focused on specific pathologies, such as melanoma [47], psoriasis [45], or occupational medicine [54]. Other studies have addressed the perspectives and issues of incorporating TD and/or AI into the *health domain* [48,49,50,51,52,53], also with reference to mHealth [51] and to opportunities in disadvantaged countries [50].

Table 2 reports a sketch of the data synthesis of the overview of the reviews.

#### 3.3.1. The Impact of the COVID-19 Pandemic

It is recognized that the COVID-19 pandemic has represented a real driving force for AI and TD, as reported in five reviews [42,43,44,46]. Lee et al. [42] reported how the mobile teledermoscopy (mTD) may represent a strategic tool between the physicians, the general practitioners, and the citizen patients. People can monitor a suspicious skin lesion previously detected at a visit or self-detect suspicious lesions and transmit images for the medical tele-consulting. Authors reported that several Apps based on AI allow this. The authors concluded that this represents an important perspective in the post COVID-19 era; however, it must be taken into account that these Apps apply AI to assess medical images, but the AI algorithms are not specific for dermoscopy, creating problems of diagnostic accuracy. Perrone et al. [43] recalled the contribution of technological innovation during the COVID-19 pandemic in different sectors of the *health domain*. They remembered that, among these sectors there is also that of dermatology, where technological innovation has allowed for the sharing and rapid detection of presumptive cutaneous signs and symptoms of COVID-19 in the context of a drastic reduction of face-to-face visits and with the saving of costs, time, and resources. In line with the two previous studies, there is the review by Goldust et al. [44] focused on the development of AI during the COVID-19 pandemic, with reference to dermatology. They showed that TD and AI were used strategically to discern six clinical dermatological patterns with a probable prognostic connection to COVID-19: (1) the livedo reticularis racemosa-like; (2) the purpuric vasculitic pattern; (3) the chilblain-like acral pattern; (4) the confluent erythematous/maculopapular/morbilliform rash; (5) the urticarial rash; and (6) the papulovesicular exanthem. The authors remarked that there is support of the scientific societies, both in the use of and in generating further medical knowledge around this. Mbunge et al. [46] focused on the technological efforts to face COVID-19 in South Africa. They remarked that among all the technologies, TD and AI also made an important contribution to the battle against COVID-19.

#### 3.3.2. Opportunities in the Field of the Melanoma, Psoriasis, and Occupational Care

Three studies specifically focused on three particular sectors regarding pathologies [45,47,54].

Havelin et al. [45] focused on psoriasis care with three points of view: applications with mobile phones, TD, and AI. They also searched for specific Apps for “psoriasis” on the website www.appannie.com (accessed on 25 April 2023). This review highlighted the presence of a high number of Apps designed to help and guide to discern and/or monitor the pathology. Authors also highlighted some criticalities: only one App had followed a validation testing; very few Apps were clinically tested; and very few Apps made published data available.

Skudalky et al. [47] focused on melanoma in response to rising rates of melanoma worldwide. They reported the novel noninvasive melanoma detection techniques useful in the early detection of the pathology and provided guided advice on the use of the different noninvasive techniques in clinical practice related to melanoma including AI.

Elsner et al. [54] remarked that both the SaF TD and the RT TD could support specialists in occupational medicine, both in the preventive care and in the cancer screening. They concluded that: TD might provide important support both in the prevention and monitoring of occupational skin pathologies and a strategic role could be played by the Apps, including AI software in the self-monitoring of workers with a high-risk job position.

#### 3.3.3. Perspectives in the Health Domain

It is strategic to consider the aspects of integration in the *health domain* in terms of perspectives, opportunities, and problems.

This theme has been touched upon, albeit sometimes in a nuanced way, by all 13 review papers [45,46,47,48,49,50,51,52,53,54].

Six studies have addressed this [48,49,50,51,52,53] from different perspectives with a wide angle of view.

Young et al. [52] reviewed dermatological applications of deep learning and analyzed the current opportunities and problems of the related applications. They categorized the applications into three fields: (i) TD, with the inclusion of triage; (ii) the augmentation of the clinical assessment in the visits in presence; and (iii) dermatopathology. The authors also remarked on the importance of the use of specific standardized metrics for the assessment of the performance of the models, as well as the need to face both equity and ethical issues when applying these tools in clinical use.

Pasquali et al. [53] highlighted the importance of TD as a valid alternative to face-to-face visits, and, in general, as a valid tool for tele-consultation, tele-education, second opinions, and remote monitoring. They also discussed the potential of artificial intelligence combined with TD techniques and medical imaging theory. It has also opened up potential legal and ethical issues.

Greis et al. [50] highlighted the advantages of TD use in countries with limited resources and with complex territories, such as those in Africa. They reported that currently there are several projects on TD active in Africa with an increasing use of AI to provide support for doctors. The authors remarked on the importance of increasing the volume of medical data to improve the performance of AI models and systems. The participation of local healthcare providers and of the global dermatological field is, according to the authors, strategic to reach this point.

Blum et al. [51] remarked on the well-known advantages of TD and AI and particularly focused on the potential disadvantages and risks of AI use, as well as on the perspectives on mHealth. They categorized the disadvantages and risks of AI use into: (1) the lack of mutual trust generated by the diminished patient–physician direct relationship. (2) The additional time needed to assess the benign lesions. (3) Absence of enough medical knowledge to detect incorrectly classified AI decisions. (4) The need to contact again the patient rapidly in the case of not correct AI classification. (5) Medico-legal issues due to lack of regulation in this field. (6) The problem of the reimbursement. (7) The Apps based on AI are currently not able to provide adequate assistance based on an image of a positive test for a cancer skin lesion.

They reported the requirements for user satisfaction for *mHealth*: Apps can be applied correctly if the quality of images is adequate, the patient’s data can be uploaded easily, data exchange allows for both the image upload and the results download, and medical reimbursement and medico-legal aspects have been disclosed.

Uppal et al. [49] particularly focused on TD. They reported the well-known advantages while also remarking that TD is not useful in monitoring chronic conditions, as this requires a continuous follow up and tuning of the therapy. They also highlighted that mTD has an increasing potential in the *health domain*. It is low cost, practicable, and user-friendly. They concluded with the recommendation to expand the applications in more fields and to focus more on the investigation of the role of AI and in the expertise in the assessment of the images exchanged by means of TD.

Tognetti et al. [48] made a map point of the state of the art advances in TD, both from a technological and clinical point of view, including the role of AI and of the web platforms. They highlighted the enormous advantages. The map point also showed the current limits of the employment of the technology. The first limit is the absence of clear regulations for TD practice. They reported the current lack of a common and clear European regulation for practicing TD, while also comprehending issues of cybersecurity. The second limit is represented by the need to design efficient workflows and plans allowing for better spread and interaction among the different actors. The third limit is a consequence of the previous two; it is the absence of agreement initiatives, with particular reference to position statements and guidelines.

## 4. Discussion

The topic investigated in the overview, relating to the integration of TD with AI, is particularly complex. In fact, the two technologies historically show, also separated, important complexities in the integration with the *health domain*. *First*, TD inherits the problems of *telemedicine, e-Health,* and *mHealth*. These problems, such as the regulation, were only patchily overcome during the COVID-19 pandemic [55,56]. *Second*, AI has a very recent history connected to its integration with digital health imaging (see, for example, the AI in digital radiology and in digital pathology [10]) and with dermatology itself [20,21]. Like any new technology, AI needs to be accepted and standardized in the scope of the applications [32,33,34,48,51,52,53].

AI is a large container of technological solutions that aims to make machines learn from their experiences, adapt to new inputs and changes quickly, and perform tasks with human logic. Surely dermatology can benefit from all the components of AI. Many promising solutions applied in a standalone way can be integrated with telemedicine, expanding the potential. In this way, many applications of dermatology will be able to have a wider extension on the territory, incorporating disadvantaged areas, and improving the aspects of service equity in the *health domain*. Many promising uses of AI, ranging from monitoring the efficacy of drugs to even the impact of diet in dermatology, will benefit from an integration with telemedicine [57,58,59,60]. For reasons of clarity, it is appropriate to report some of these experiences to make it clear how AI is integrating at 360 degrees with dermatology and how telemedicine could play a further driving role [57,58,59,60]. For example, important perspectives have been glimpsed in the use of AI to predict drug efficacy in dermatology [57], applying, for example, two different approaches based on artificial neural networks (ANN)s: the auto-contractive map (Auto-CM) and an unsupervised ANN to study how these variables cluster, and a supervised ANN and Training with Input Selection and Testing (TWIST), to build the predictive model. The use of an artificial neural network to identify patient clusters in a large cohort of patients with melanoma was proposed in [58]. It allowed for a simultaneous analysis of costs and clinical characteristics, both relevant aspects for the health service in the *health domain*. The study reported in [59] is very significant, as it is also linked to aspects of therapy, specifically, to aspects of radiotherapy. This study highlighted how ANNs allow for response prediction in squamous cell carcinoma of the scalp treated with radiotherapy [59]. A very important example of the use of AI in relation to acne (a very common problem affecting young people) is reported in [60]. This study confirmed the link between several dietetic items and acne, highlighting the complex interconnection between dietetic factors and acne, which must be taken into serious consideration by dermatologists [60].

### 4.1. Added Value of the Review

Several studies based on scientific articles and reviews have separately addressed the introduction of AI and TD into dermatological practice. The narrative reviews are particularly useful in relatively young sectors in the processes of MK construction. In fact, they are flexible in terms of the search strategy and selection criteria, and do not require the inclusion of a formal quality assessment, in comparison to the systematic reviews. Systematic reviews are an important tool, but sometimes they are ineffective to apply in very young scientific fields where there is still little MK. This narrative review intended to make a contribution by investigating the introduction of the two technologies jointly. In particular, the proposed overview aimed to provide added value by acting as a connector between all the specific issues addressed in this field to date.

### 4.2. Interpretation of Results

The research conducted has revealed how this line of studies is particularly recent [19]. Furthermore, an important driving force was represented by the technological innovations that occurred during COVID-19 [42,43,44,46]. TD and AI contributed to maintaining both a high quality of care during the pandemic and in the monitoring and detection of some skin pathologies with prognostic probabilities of the COVID-19 disease [48]. This initiated the diffusion of technology which affects all the actors involved in dermatology, including the citizen, and presents opportunities, perspectives, peculiarities, and bottlenecks.

#### 4.2.1. Opportunities and Perspectives

The overview showed various opportunities and perspectives in this area. Generally, all the studies converged on the opportunities and general perspectives of TD and AI in improving the quality of service overall. Specifically, they showed that TD and AI could improve: the quality of care, healthcare processes, the cost savings, the stress in healthcare facilities, citizens’ satisfaction, and the care, even in disadvantaged realities (as shown, for example, in a study conducted in Africa [50]). Several studies have documented opportunities in different sectors of dermatology. One of TD and AI ‘s greatest strengths may be its utility as a triage and monitoring tool. This is critical in the early detection of skin cancer, as it can reduce the number of unnecessary referrals, the wait times, and the cost of providing and receiving dermatological care [23,47,54]. Other sectors of interest in the application of AI have also been highlighted, such as that for sun perilesions [41] and in occupational medicine [54]. In the latter sector of occupational medicine, the authors highlighted how TD and AI might provide important support both in the prevention and monitoring of occupational skin pathologies. They recognized a strategic role of the Apps including AI software in the self-monitoring of workers with a high-risk job position. It is precisely the Apps and their integration into *mHealth* that present great opportunities for the self-care of a citizen who can remotely integrate with the health system [30,31,45,49,51] with high satisfaction, as per the specific survey [31].

The opportunities and prospects for integration into the *health domain* are promising based on acceptance studies conducted through surveys [35,36]. In these studies, conducted through specific surveys, emerged a balanced view on the use of AI (which is considered complementary to human decision making) and an interesting, positive opinion on the opportunity. Important opportunities for TD and AI are also perceived in image quality control. Here, it has been seen that through appropriate tools and models [27,28,29], it is possible to carry out targeted assessments to guarantee images with parameters suitable for diagnostics.

The integration of the TD and AI has many elements in common with digital radiology and digital pathology. Among these elements, as highlighted in this overview, we find: the improvement of the quality of the service (in all its components), the quality control of the images [27,28,29], the revision of the workflows, and the need to tackle in a decisive and incisive way the medico-legal, ethical and regulatory issues [48,52,53]. We also find these fields of investigation in digital radiology and digital pathology [18,61]. Even the way of proposing specific and non-standardized surveys in the literature to evaluate acceptance [35,36] is something in common between these three disciplines [61,62]. However, we should consider that dermatology is more patient oriented. In other words, the patient (citizen) is more involved with providing a picture of a lesion/wound. With pathology and radiology, physicians are looking at images provided by a lab, clinic, imaging center, etc.

The real peculiarity of TD and AI, inherited by the dermatology is the possibility of citizens directly using Apps in *mHealth* to access virtual or remote diagnosis and self-care [30,31,45,49,51], in a decidedly stimulating way [31]. This certainly opens many opportunities for TD and AI but also new problems. In both digital radiology and digital pathology, the virtual interaction is, in fact, reserved exclusively for the professionals in a working network connection environment. Table 3 reports in brief the emerging advantages and opportunities of the TD and AI in dermatology.

#### 4.2.2. Emerging Problems and Bottlenecks

The overview also showed some important problems and bottlenecks to overcome.

*First*, these rapid developments to ensure a better fruition by all should be accompanied by [48]:

(a) targeted agreement initiatives, with reference to position statements and guidelines;
(b) the design of both efficient workflows and plans, allowing for better spread and interaction among the different actors.

*Second*, synergistic regulatory initiatives at an international and local level are indispensable for these complex systems, which suffer in this area from the same shortcomings of other systems in other sectors [61].

*Third*, it is necessary to focus on the peculiarities of the use of Apps in TD and AI in the hands of citizens. It is important to: clarify the validation processes [45], pay attention to the aspect of cybersecurity, and carefully focus on the system design, standards, and the completeness of the features proposed to citizens [32,33,48,51].

*Fourth,* in both *eHealth* and *mHealth* applications, before applying TD and AI, it is necessary to provide for the disclosure of all medico-legal (including reimbursement) and ethical aspects.

*Fifth,* with reference to Apps for citizens, it is necessary to avoid the risks of so-called self-care (i.e., the citizen controls/monitors themselves in a pathological way and/or does everything by themselves without consulting experts). Table 4 outlines, in brief, the emerging problems and the bottlenecks.

### 4.3. Limitations

This narrative overview has limitations. This review considered papers written in English and two in German (available via PubMed). The reviews in different languages not available in these databases were not considered. PubMed and Scopus databases were consulted, and only peer-reviewed papers were considered in this review. Databases at a local/national level were not consulted.

## 5. Conclusions

There is great enthusiasm for the introduction of AI in the healthcare field and, in particular, in TD.

The pandemic provided an important impetus for the integration of AI in TD, as in other fields. The study conducted a narrative review investigating the opportunities, the perspectives, and the bottlenecks of this integration.

Applications of this integration have been identified in various pathologies and in quality control, both in *eHealth* and *mHealth*. Many of these applications are based on Apps used by citizens for self-care in *mHealth*. This is an important peculiarity of TD that differentiates it from other imaging applications (such as digital radiology and digital pathology) with new opportunities but also open questions.

A generalized enthusiasm has been registered which converges on the opportunities and general perspectives of the TD and AI in improving the quality of the service overall, and, specifically, in the quality of care, in optimizing the healthcare processes, in minimizing costs, in reducing stress in healthcare facilities, and in making citizens, now at the center, more satisfied.

However, critical issues have emerged. Particular attention must always be paid on the Apps in the hands of the citizen, in the validation, the design, the proposal of functions, and in the safety requirements for completing the flow image detection up to the diagnosis, in terms of standardization and cybersecurity. International and national regulations have not yet been stabilized. It is always necessary to proceed with clarifications of the medico-legal and ethical aspects, before using a specific tool in a connection. In general, to ensure a better fruition by all, the following should accompany TD and AI: targeted agreement initiatives, with particular reference to position statements, guidelines, and consensus initiatives, and the design of both efficient workflows and plans, allowing for better spread and interaction.

## Figures and Tables

**Table 1:** Table supporting the data synthesis of overview of scientific papers.

	Brief Summary of the Research
[8]	Great opportunities in using TD and AI for remote diagnosis of skin lesions have been highlighted.
[21]	Three key elements in TD and AI have been identified: the role and expectations of AI, the applicability of imaging techniques, and the developments in many applications. These elements offer great help in health and define routine models.
[22]	Important prospects for TD and AI have been identified in virtual diagnosis, research, and development of advanced machine learning models for prognosticating dermatoses and classification, detection, and diagnosis.
[23]	A proposed unified CAD model approach using AI showed to provide both lesion segmenting and classifying at the skin level with high performance and accuracy in imaging parameters.
[24]	External testing was demonstrated essential for regulating the development of machine learning models in primary care settings.
[25]	Important steps in the evolution of the tools that can be used in TD and AI have resumed. In this area, together with new technologies, new approaches and methodologies must coexist with historically consolidated methods.
[26]	An automatic TD and AI tool improved the diagnosis of skin lesions by non-dermatologists, such as nurse practitioners or primary care specialists, with high potential for improving healthcare quality in dermatology.
[27]	Quality assessment tools based on AI could identify criticalities in image quality, demonstrating performance similar to that of dermatologists and improving the TD process.
[28]	A tool demonstrated a quality detection performance like that of dermatologists, allowing an improvement of the TD process.
[29]	AI showed a leading role in ensuring the usefulness of a certain image in TD, and that different solutions for quality assessment require tuning for specific TD applications.
[30]	The use of mobile applications demonstrated the potential to improve the participation in research projects during the pandemic. *Pros* and *cons* have been highlighted.
[31]	A study on the assessment of user satisfaction with a smartphone-compatible application using an AI algorithm was proposed. The results highlighted both a high satisfaction and that the Apps were promising for all the actors of the *health domain*.
[32]	A study in two parts was proposed. The first part highlighted that image postprocessing capabilities varied greatly based on the user and the intended function. Second, technology standards were not always implemented or reported. Critical issues were also detected on the consent procedure and on other aspects of privacy and data confidentiality.
[33]	The second part of the study (se immediately above) highlighted that: the choices of different technical applications of image acquisition were hardly ever proposed in the tools, and users of these applications should consider which tools had standardized functionalities capable of improving the image quality.
[34]	The problem of standards was addressed in a study with specific reference to the reality of the Canadian nation.
[35]	A survey highlighted the belief that after COVID-19 this technology: could be of aid; will not replace but support professionals; and needed specific regulatory measures.
[36]	A survey concluded that the technological innovations, including the use of AI and TD, could contribute to improving and enlarging the use of dermoscopy among general practitioners.
[37]	An AI algorithm was evaluated in a telemedicine setting configured for TD where patients provided images via telemedicine. The study demonstrated that this configuration of the healthcare process was promising both in triage and in the assessment of skin lesions.
[38]	An applied specific tool had very high specificity and sensitivity in TD and AI applications, showing potential to assist the clinical decision-making process.
[39]	The accuracy of two countries in the national screenings in a certain dermatological field was compared. It was deduced that TD and AI could aid in speeding up the early diagnosis of melanoma, making the process easier and more economical without stressing the structures of the health systems.
[40]	A project in progress was summarized. The aim was to: compare the decision taken by an AI-based classifier with that of the teledermatologist, the histopathologist, and other key professional figures. Consequently, to assess the impact on diagnostic and process management decisions including time, costs, personnel, and activities.
[28]	Applications of AI in Dermatology Image Quality Assessment was discussed. AI was was find useful to ensure the clinical utility of images for remote consultations and clinical trials.
[41]	Applications and perspectives on sun damage perilesions were reported, showing strengths and weakness of the applications.

**Table 2:** Table supporting the data synthesis of the *overview of the reviews*.

	Brief Summary of the Research
[42]	The use of mobile teledermoscopy (mTD) as a tool to improve communication and diagnosis between physicians, general practitioners, and patients was described. Patients could monitor suspicious skin lesions and send images for teleconsultation with the help of various AI-based Apps. It was highlighted that while this technology offered a promising solution in the post-COVID era, it should be noted that AI algorithms used in these Apps were not specific to dermoscopy, which could lead to problems with diagnostic accuracy.
[43]	The role of technological innovation in various sectors of healthcare during the COVID-19 pandemic, including dermatology, was highlighted. The review reported that technology had enabled the sharing and rapid detection of cutaneous signs and symptoms of COVID-19, resulting in reduced face-to-face visits and cost and time savings
[44]	The development of AI during the COVID-19 pandemic with reference to dermatology was addressed. Research showed that the use of TD and AI was strategic to discern six clinical dermatological patterns with a probable prognostic connection to COVID-19.
[45]	The application of the TD and AI in psoriasis was investigated highlighting: the presence of a high number of Apps designed to help and guide to discern and/or monitor the pathology, and criticalities due to scarce App testing, validation, and publication data.
[46]	The technological efforts to face COVID-19 in South Africa were remarked on in the review. It was reported that, among all the technologies, TD and AI provided an important contribution to the battle against COVID-19.
[47]	The application of TD and AI in melanoma was investigated, highlighting that the novel noninvasive melanoma detection techniques were useful in the early detection of the pathology, as well as several useful pieces of advice on the use of different noninvasive techniques in clinical practice.
[48]	It was reported a map point on the state of the art advances in TD and AI from both technological and clinical perspectives, including the role of AI and web platforms. The review highlighted the benefits of TD and AI, but also identified three limits to its implementation: the absence of clear regulations for TD and AI practice, the need for efficient workflows and plans to better interact with different actors, and the absence of agreement initiatives such as position statements and guidelines.
[49]	The review focused on mobile TD (mTD) and its advantages in the *health domain*, such as low cost, practicality, and user-friendliness. mTD was considered not useful for monitoring chronic conditions that required continuous follow up and therapy tuning. Recommendations include expanding the applications of mTD in other fields and focusing more on the investigation of the role of AI and image assessment in TD.
[50]	The review discussed the advantages of TD and AI in countries with limited resources and complex territories such as in Africa. It highlighted the importance of increasing the volume of medical data to improve the performance of AI models and systems.
[51]	The review faced the potential disadvantages and risks of using AI in TD, particularly in the context of mHealth. The study recommended that satisfactory use of AI in mHealth required high-quality images, easy patient data upload, data exchange for both image upload and results download, as well as robust cybersecurity, and full disclosure of medical reimbursement and medico-legal aspects.
[52]	The study reviewed dermatological applications of deep learning and categorized them into three fields: TD, clinical assessment augmentation, and dermatopathology. It stressed the importance of using standardized metrics for performance assessment of deep learning models and for addressing equity and ethical issues when applying these tools in clinical use.
[53]	The review highlighted the importance of TD as a valid alternative to *face-to-face* visits and a useful tool for tele-consultation, tele-education, second opinions, and remote monitoring. It was also discussed the potential of combining artificial intelligence with TD techniques and medical imaging theory and the legal and ethical issues surrounding these applications.
[54]	The potential of both store and forward TD and real-time TD in supporting specialists in occupational medicine for preventive care and cancer screening was reported. The study concluded that TD could play an important role in the prevention and monitoring of occupational skin pathologies and that Apps, including AI software, could have a strategic role in the self-monitoring of workers in high-risk job positions.

**Table 3:** Advantages and opportunities of TD and AI in dermatology.

	Advantages/Opportunities
*1*	Improved quality of care, healthcare processes, and cost savings.
*2*	Reduced stress in healthcare facilities.
*3*	Increased citizen satisfaction and care, also in disadvantaged realities.
*4*	Early detection of skin cancer, leading to a reduction in unnecessary referrals, wait times, and cost of care.
*5*	Opportunities in specific sectors, such as the skin cancer, the sun perilesions, and the occupational medicine.
*6*	Utility as a triage and monitoring tool.
*7*	Integration with mHealth for self-care and remote diagnosis.
*8*	Balanced view on the use of AI and positive opinions on the opportunities.
*9*	Image quality control through appropriate tools and models.
*10*	Similar elements of process improvement, quality control, workflow revision as in digital radiology and digital pathology.

**Table 4:** Problems and bottlenecks of TD and AI in dermatology.

	Problem/Bottlenecks
*1*	Targeted agreement initiatives, with reference to position statements and guidelines (including regulations and ethics), to accompany the rapid developments of TD and AI (especially in mHealth) are strongly needed
*2*	Efficient workflows and plans are necessary to allow for better spread and interaction among the different actors involved in the use of TD and AI.
*3*	Synergistic regulatory initiatives are indispensable, both at an international and local level, to address the shortcomings of complex systems in TD and AI, as well as in other sectors.
*4*	It is crucial to focus on the peculiarities of the use of Apps in TD and AI in the hands of citizens. This includes clarifying validation processes, paying attention to cybersecurity aspects, and carefully focusing on system design, standards, and completeness of features proposed to citizens.
*5*	Before applying TD and AI in eHealth and mHealth applications, it is necessary to provide for the disclosure of all medico-legal (including reimbursement) and ethical aspects.
*6*	The risks of self-care, where citizens control/monitor themselves in a pathological way or without consulting experts, must be avoided when designing Apps for citizens.

## Data Availability

Not applicable.
